# Hormones and immunity in cancer: are thyroid hormones endocrine players in the microglia/glioma cross-talk?

**DOI:** 10.3389/fncel.2015.00236

**Published:** 2015-06-23

**Authors:** Cristiana Perrotta, Clara De Palma, Emilio Clementi, Davide Cervia

**Affiliations:** ^1^Department of Biomedical and Clinical Sciences “Luigi Sacco” (DIBIC), Unit of Clinical Pharmacology, National Research Council-Institute of Neuroscience, University Hospital “Luigi Sacco”, Università di MilanoMilano, Italy; ^2^Scientific Institute IRCCS Eugenio MedeaBosisio Parini, Italy; ^3^Department for Innovation in Biological, Agro-food and Forest Systems (DIBAF), Università della Tuscia, Largo dell’Università sncViterbo, Italy

**Keywords:** thyroid hormones, microglia, macrophages, glioma, tumor microenvironment, hypothyroidism, tumor growth

## Abstract

Accumulating evidence indicates that the endocrine and immune systems engage in complex cross-talks in which a prominent role is played by thyroid hormones (THs). The increase of resident vs. monocyte recruited macrophages was shown to be an important effector of the TH 3,3′,5′-Triiodo-L-thyronine (T3)-induced protection against inflammation and a key role of T3 in inhibiting the differentiation of peripheral monocytes into macrophages was observed. Herein, we report on the role of T3 as a modulator of microglia, the specialized macrophages of the central nervous system (CNS). Mounting evidence supports a role of microglia and macrophages in the growth and invasion of malignant glioma. In this respect, we unveil the putative involvement of T3 in the microglia/glioma cell communication. Since THs are known to cross the blood-brain barrier, we suggest that T3 not only exerts a direct modulation of brain cancer cell itself but also indirectly promotes glioma growth through a modulation of microglia. Our observations expand available information on the role of TH system in glioma and its microenvironment and highlight the endocrine modulation of microglia as an important target for future therapeutic development of glioma treatments.

## Introduction

The specialized macrophages of the central nervous system (CNS), namely microglia, constitute 5–20% of total glial cells (Ransohoff and Perry, 2009; Kettenmann et al., 2011; Saijo and Glass, 2011). The lineage relationship between microglia and peripheral macrophages is well established (Yang et al., 2010; Saijo and Glass, 2011); it has been recently suggested that microglia originate from macrophages migrating into the CNS during early embriogenesis and that microglial cell population can locally expand in CNS (Ginhoux et al., 2010; Saijo and Glass, 2011). Our understanding of the key factors and molecular mechanisms responsible for microglia development and function is however still incomplete. In a healthy environment, resting microglia displays low expression levels of inflammatory molecules, but when activated, microglial cells abandon their ramified surveiling morphology, become ameboid, acquire phagocytic functions and migrate to the injured site to release inflammatory molecules (Polazzi and Monti, 2010; Saijo and Glass, 2011). Generally, microglial cells act as the primary responding cells for infectious and traumatic stimuli although their activation may also result in pathological forms of inflammation that contribute to the progression of neurodegenerative diseases (Glass et al., 2010; Perry et al., 2010; Saijo and Glass, 2011; Assi et al., 2013).

Studies of peripheral macrophages have led to the development of the concept of two different macrophage activation states, i.e., the “classically activated” (M1) and “alternatively activated” (M2) ones (Murray and Wynn, 2011; Sica and Mantovani, 2012). The “classically activated” macrophages express pro-inflammatory cytokines, mediate defense of the host from a variety of bacteria, protozoa and viruses, and have roles in anti-tumor immunity. The “alternatively activated” macrophages have anti-inflammatory, pro-tumoral function and regulate wound healing. It is generally assumed that macrophages activation *in vivo* represents extreme of a continuum in a universe of activation states and mixed phenotypes and coexistence of cells in different activation states have been observed in preclinical/clinical conditions (Sica and Mantovani, 2012). These concepts might also be applicable in the case of microglia which has activation states similar to that of macrophages and exhibits functional plasticity during activation states (Saijo and Glass, 2011). However, the associations between distinct activation states and pathology are less well defined and may differ from those of macrophages in peripheral tissues (Ghosh and Chaudhuri, 2010; Yang et al., 2010; Saijo and Glass, 2011; Wei et al., 2013).

Similarly to other tissues, brain cancers are complex ecosystems composed of many interacting elements. The communication between the tumor cells and the surrounding cells helps to drive the process of tumor progression and the shaping of its complexity. Increasing evidence indicates that what is happening inside the tumor cell occurs also under exogenous stimuli arising around tumor cells (Albini and Sporn, 2007; Joyce and Pollard, 2009; Charles et al., 2012; Goubran et al., 2014; Klemm and Joyce, 2015). Beyond cancer cells, microglia, astrocytes, the extracellular matrix and soluble factors influence the tumor invasion, angiogenesis, cell proliferation/apoptosis also having profound effects on the efficacy of cancer therapies (Albini and Sporn, 2007; Joyce and Pollard, 2009; Charles et al., 2012; Goubran et al., 2014; Klemm and Joyce, 2015; Gutmann, 2015). In the case of malignant gliomas, a primary CNS cancers arising from glial cells, our understanding of the role of microenvironmental cells has lagged behind the discovery that monocytes are the most likely source of all brain macrophages and that microglia and macrophages may account for a large amount of total cell populations in brain tumors (Watters et al., 2005; Saijo and Glass, 2011; Gutmann, 2015). In this regard, glioma tissue shows high levels of infiltrating microglia, localized diffusely throughout the tumor, rather than to the areas of necrosis (Yang et al., 2010; Charles et al., 2012). Although once previously thought to play an anti-tumorigenic role, microglia has recently emerged as important element in the progression and growth of glioma through diverse mechanisms (Ghosh and Chaudhuri, 2010; Yang et al., 2010; Saijo and Glass, 2011; Zhai et al., 2011; Charles et al., 2012; Jacobs et al., 2012; Wei et al., 2013; da Fonseca and Badie, 2013; Gutmann, 2015). Glioma-associated microglia produce plenty of cytokines, chemokines, interleukins, and growth factors, which can either shape a more permissive tumor microenvironment or directly trigger glioma cell growth and invasion. In particular, by inducing new blood vessel formation and/or changes in the extracellular matrix microglia may create indirectly a supportive soil that further enhances glioma growth or invasion. Alongside microglia-released soluble factors may increase directly glioma stem cell or astrocytoma cell proliferation, survival, and/or invasion. In addition, glioma-infiltrating microglial cells appear incapable of inducing an effective anti-tumor T cell response, strongly supporting the fact that microglias promote tumor growth by facilitating immunosuppression of the tumor microenvironment. Of notice, glioma cells may over-rule the normal defensive role of microglial cells and confine them into an immune-depressive boundary. In this context, the elucidation of the microglia-glioma ecosystem can provide useful information for manipulation of the glioma microenvironment in a therapeutic perspective, i.e., to generate a specific and durable anti-glioma immune response.

## Thyroid Hormones and Macrophages/Microglia

The endocrine and immune systems engage in complex cross-talks. Hormones and endocrine transmitters bind to immune system cells, thus modifying immune cell functions and tuning immune responses (Dorshkind and Horseman, 2000; Kelley et al., 2007; Barnard et al., 2008; Butts and Sternberg, 2008; Rivest, 2010; Carlton et al., 2012). In this respect, growing evidence indicates that the thyroid hormones (THs) 3,3′,5′-Triiodo-L-thyronine (T3) and L-thyroxine (T4) are important modulator factors of immune cells, including peripheral macrophages (Khansari et al., 1990; Rosa et al., 1995; Forner et al., 1996; Rittenhouse and Redei, 1997; Ortega et al., 1999; Dorshkind and Horseman, 2000; El-Shaikh et al., 2006; Klecha et al., 2006; Mascanfroni et al., 2008; Mazzoccoli et al., 2010; De Vito et al., 2011; Chen et al., 2012). Recently we identified a homeostatic link between T3 and the pathophysiological role of macrophages (Perrotta et al., 2014). In particular, our *in vitro* results indicate a negative role of T3 in triggering the differentiation of mouse circulating monocytes into macrophages. T3 was also shown to induce macrophages to display a “classically activated” signature, as revealed by the expression analysis of surface proteins and cytokine release, as well as the experiments on cell migratory ability (chemotaxis) and phagocytosis. Interestingly, the analysis of gene markers in macrophages treated with T3 revealed a somehow “classically activated”/“alternatively activated” mixed phenotype thus suggesting that the switching induced by T3 is very complex. *In vivo* results demonstrated that circulating T3 increased the content of the resident macrophages in the mouse peritoneal cavity while reducing the content of the recruited monocyte-derived cells. Additionally, T3 significantly protected mice against endotoxemia: decreased T3 levels increased the recruited (potentially damaging) cells while the restoring of T3 levels decreases recruited and increases resident (potentially beneficial) cells (Perrotta et al., 2014). Although macrophages were historically considered to be derived from the blood monocyte reservoir, numerous studies have since demonstrated that, under steady-state conditions, resident tissue macrophage populations are largely maintained through local proliferation (Yona and Jung, 2010). Inflammatory insults, however, result in the rapid recruitment of blood-borne precursors to the respective tissue macrophage compartment (Yona and Jung, 2010). In this line, our data suggest that T3 contributes to limit inflammation by promoting the proliferation of peritoneal macrophages *in situ*, while inhibiting the potentially damaging cell recruitment from monocyte cell pools, in a context not fully explained by the “classically activated”/“alternatively activated” framework (Perrotta et al., 2014).

The influence of thyroid imbalance on microglial development was firstly identified in 2001 when hypothyroidism was found to slow markedly the progressive elaboration of microglial processes in the developing rat forebrain and increases in T3 levels accelerate them (Lima et al., 2001). In addition, *in vitro* and *in vivo* analyses revealed that T3 increases the number of microglia cell bodies, promotes microglia survival (but not the proliferation) and enhances growth of their processes (Lima et al., 2001). These results indicate that THs promote the growth and morphological differentiation of cortical microglia during development. Accordingly, it has been recently shown that hypothyroidism prominently reduces the processes of microglia in the hippocampus of diabetic rats (Nam et al., 2013).

## Thyroid Hormones at the Interplay Between Microglia and Glioma Cells

**Figure 1:**
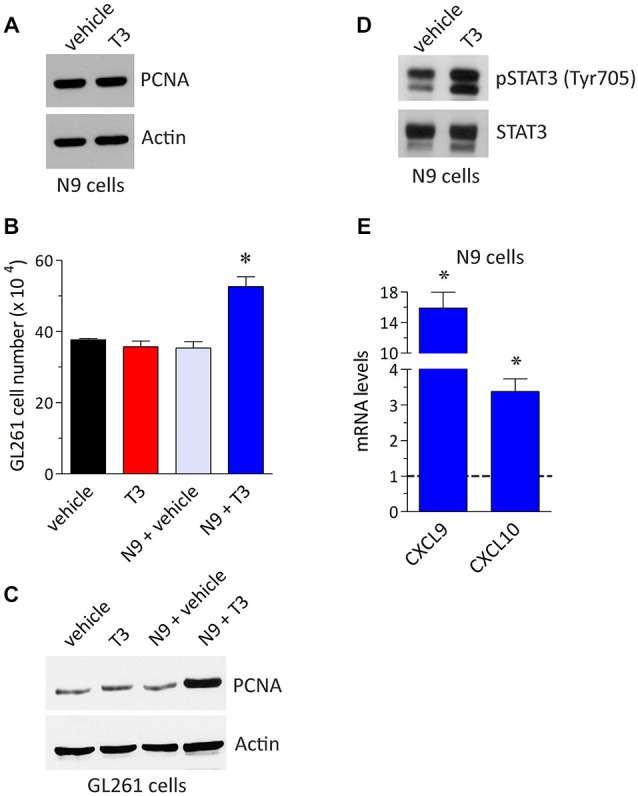
**T3 induces glioma cell growth by a direct action on microglia. (A)** Expression of the proliferation marker proliferating cell nuclear antigen (PCNA) in N9 cells plated in the absence and in the presence of T3 (1 μM, 24 h). The Western blot analysis was performed as described previously (Armani et al., 2007; Cervia et al., 2007, 2013; Bizzozero et al., 2014; Cazzato et al., 2014; De Palma et al., 2014; Perrotta et al., 2014) using the mouse monoclonal anti-PCNA (PC-10) and the goat polyclonal anti-actin (I-19) (internal standard) primary antibodies (Santa Cruz Biotechnology, Dallas, TX, USA). The image is representative of results obtained from three different experiments (*n* = 3). **(B)** GL261 cell number in co-culture experiments. The experimental setting was in agreement with a previous report (Zhai et al., 2011), with minor corrections. Briefly, GL261 cells were seeded in the bottom wells of Costar transwell plates (24-mm diameter insert, 0.4 μM pore size, polycarbonate membrane; Corning Life Sciences, Corning, NY, USA) with or without N9 cells in the top wells (1:1 N9:GL261), both in the absence or in the presence of T3 (1 μM). Cell concentration after 24 h cultures was measured by counting trypan blue-excluding cells with TC20 Automated Cell Counter (Bio-Rad, Hercules, CA, USA), as described previously (Cervia et al., 2013; Perrotta et al., 2014). Each histogram represents the data obtained from 3–6 different experiments (*n* = 3–6). The results were expressed as means ± SEM. *P < 0.001 vs. the other values, using one-way ANOVA followed by the Tukey’s multiple comparison post-test (GraphPad Prism; GraphPad Software, La Jolla, CA, USA). **(C)** Western blot analysis of PCNA in GL261 cells co-cultured as described above. The image is representative of results obtained from three different experiments (*n* = 3). **(D)** STAT3 phosphorylation in N9 cells plated in the absence and in the presence of T3 (1 μM, 24 h). The Western blot analysis was performed using the rabbit polyclonal anti-phospho STAT3 (Tyr705) and the anti-STAT3 primary antibodies (Cell Signaling Technology, Danvers, MA, USA). The image is representative of results obtained from three different experiments (*n* = 3). **(E)** Real-time quantitative PCR experiments of mRNA levels for CXCL9 and CXCL10 in N9 cells in the presence of T3 (1 μM, 24 h). Experiments were performed as previously detailed (Cervia et al., 2008, 2012, 2013; Charles et al., 2012; Bizzozero et al., 2014; Cazzato et al., 2014; De Palma et al., 2014; Perrotta et al., 2014). Primer pairs: CXCL9, 5′-TCCTTTTGGGCATCATCTTCC-3′ (forward) and 5′-TTTGTAGTGGATCGTGCCTCG-3′ (reverse); CXCL10 5′-TCCTTGTCCTCCCTAGCTCA-3′ (forward) and 5′-ATAACCCCTTGGGAAGATGG-3′ (reverse) (Primmbiotech, Milano, Italy). Values are expressed as the fold change over control (untreated N9 cells). Each histogram represents the data obtained from three different experiments (*n* = 3) run in triplicate. The results were expressed as means ± SEM. *P* < 0.05 vs. respective control (one-way ANOVA followed by the Tukey’s multiple comparison post-test).

An aspect that is worth pursuing to understand better the interplay between the immune system and glioma is the role of the endocrine system since both contribute with an integrated action in the maintenance of the body defense against tumors. For instance, hormone dysregulations may determine the efficacy of chemo- or immuno-modulatory therapies likely affecting the tumor microenvironment (Mazzoccoli et al., 2010; ThyagaRajan and Priyanka, 2012; Armaiz-Pena et al., 2013; Goubran et al., 2014). In order to get new insight on the possible role of T3 in the regulation of microglia/glioma cross-talk we used here a retroviral-immortalized cell line, the N9 microglia line, and the GL261 murine model of malignant glioma as previously reported (Davis et al., 2006; Zhang et al., 2009, 2011; Liu et al., 2011; Zhai et al., 2011). The N9 microglia is derived from mouse brain and shares many phenotypical characteristics with primary mouse microglia, also maintaining the crucial properties of *in vivo* microglia (Stansley et al., 2012). N9 and GL261 cell lines were cultured in Dulbecco’s Modified Eagle’s Medium supplemented with 10% heat inactivated fetal bovine serum, 2 mM glutamine, 100 UI/ml penicillin and 100 μg/ml streptomycin (Euroclone, Milano, Italy) at 37°C, 5% CO_2_ in an humidified atmosphere. During treatments, cells were exposed to THs-depleted medium (Perrotta et al., 2014). T3 (Sigma-Aldrich, Saint Louis, MO, USA) was added to the cell medium for 24 h at the concentration of 1 μM, giving maximal receptor occupancy in macrophages (Perrotta et al., 2014). Parallel cultures were maintained with T3 vehicle and used as a control. As shown in the western blot experiment of Figure 1A, the levels of proliferating cell nuclear antigen (PCNA) in N9 microglia did not change in the presence of T3, further confirming that T3 was not coupled to microglia proliferation (Lima et al., 2001). We then set-up an indirect co-culture experimental procedure in which GL261 cells were plated in the bottom wells with or without N9 cells and T3 in the top wells. Using this system we observed a significant increase in GL261 cell proliferation in the presence of T3 and N9 cells when compared to GL261 with N9 only (about 49%), while T3 had no effect on GL261 cell number in the absence of N9 (Figure 1B). These results were confirmed by the analysis of PCNA protein expression (Figure 1C). Although THs (especially T4) were suggested to be a growth factor for different glioma cells *in vitro* (Davis et al., 2006; Lin et al., 2009), in our experimental settings T3 itself did not affect GL261 proliferation. Accordingly, similar concentrations of T3 did not modify PCNA levels in GL261 cells (Davis et al., 2006). In this respect, T3 effect on cell growth appears to be dependent on the type of glioma tumor cell line (Liappas et al., 2011).

The activation of signal transducers and activators of transcription 3 (STAT3) has been proposed to play an anti-tumor immunity role (Yu et al., 2014), and indeed activation of STAT3 in N9 cells increased GL261 growth (Zhang et al., 2009). Interestingly, it is becoming apparent that STAT3 is an important molecular player that allows glioma cells to promote the activity of microglia; reciprocally microglia facilitate tumor survival, growth and the spread of glioma cells (Zhang et al., 2009; Wu et al., 2010; Wei et al., 2013; da Fonseca and Badie, 2013). The inhibition of STAT3 function in tumor microglia may thus potentially be used as an immunotherapy approach for gliomas. We reported here an activatory role of T3 on STAT3 of microglia since N9 treatment with T3 resulted in elevated levels of STAT3 phosphorylation when compared to control (Figure 1D). In addition, as shown in real-time quantitative PCR experiments of Figure 1E, treatment of N9 microglia with T3 increased the mRNA expression of chemokine (C-X-C motif) ligand (CXCL) 9 and CXCL10 by 15.9 and 3.4 fold, respectively, vs. untreated control. Similar results were obtained in mouse peripheral macrophages (Perrotta et al., 2014). Chemokines constitute a significant portion of the modulatory messengers that can be released by activated microglia and interact with specific transmembrane G protein-coupled receptors (Hanisch, 2002). Of interest, both *in vitro* and *in vivo* experiments using different glioma tumors, including GL261 cells, indicated CXCL9 and CXCL10 (which bind to their endogenous receptor CXCR3) as key ligands promoting the growth of glioma (Liu et al., 2011). In this respect, different evidence indicates CXCR3 as an independent prognostic factor for glioblastoma patients and promotes an invasive phenotype (Pu et al., 2015).

Taken together our results indicate that T3 promotes GL261 glioma growth through a modulation of N9 microglia and that T3 effects involve the modulation of soluble factors released by microglia. From a mechanistic point of view, we suggest that STAT3 activation and the release of CXCL9/10 are suitable candidates to answer the question of how microglia supports glioma growth. This hypothesis, however, needs to be verified by different experimental approaches using, for example, pharmacological and/or genetic manipulations. This may also help to fully understand the signaling pathway mediating T3 actions. Indeed, STAT3 and its downstream effectors may act in parallel with different transduction mechanisms. Also, the possibility that soluble factors other than chemokines may be involved in the modulation of glioma growth cannot be excluded. At present, the pathological significance of T3-microglia-glioma axis *in vivo* remains to be established. The study of this complex issue and its molecular players appears of great interest and might highlight targets for future therapeutic development of glioma treatments based on endocrine modulation of microglia.

## Relevance of Thyroid Hormones in Glioma Therapy

There is increasing evidence that alterations in TH system are common events in cancer (Aranda et al., 2009; Moeller and Führer, 2013). However, our current understanding of the effects of THs on cancer cells reflects a rather complex picture and conflicting results mainly obtained in *in vitro* and *in vivo* animal models have also been reported. Indeed, in addition to the studies describing that THs can function as tumor suppressors, other reports support the concept that THs can enhance carcinogenesis, thus suggesting a dual role of THs (Aranda et al., 2009; Moeller and Führer, 2013).

Although no unequivocal association between thyroidal status and human cancer has been demonstrated, epidemiology and clinical studies strongly support a generalized tumor-promoting effects of THs and suggest the possibility that thyroid function/dysregulations influence the outcome of tumor therapy (Hercbergs et al., 2010; Ashur-Fabian et al., 2013; Moeller and Führer, 2013). In this respect, hypothyroidism is associated with a favorable outcome in several cancer types (Hercbergs et al., 2010; Moeller and Führer, 2013). In brain tumors, the concentration and metabolism of THs found in human tissues are altered thus suggesting that changes in circulating levels of THs may be related to malignant progression of gliomas (Nauman et al., 2004). In addition, treatment-induced hypothyroidism in glioma patients significantly improves survival and response to tamoxifen (Hercbergs et al., 2003, 2010; Moeller and Führer, 2013). Also, the successful long-term tumor response to medically induced chemical hypothyroidism in conjunction with carboplatinum chemotherapy of an adult patient with glioma was recently reported (Ashur-Fabian et al., 2013). Yet, it is still possible that hypothyroidism is only a surrogate marker for treatment efficacy and does not positively influence treatment outcome by itself (Moeller and Führer, 2013).

## Conclusion and Outlook

Malignant gliomas are aggressive, highly invasive, and neurologically destructive tumors considered to be among the deadliest of human cancers. Three decades of intensive research and a variety of chemotherapy regimes, radiotherapy and surgical approaches have been trialed and investigated, however the prognosis for patients with malignant glioma has not changed significantly (Desjardins et al., 2005; Taylor, 2010; Talibi et al., 2014). This has stimulated active research in multiples areas and the advent of new treatment strategies.

**Figure 2:**
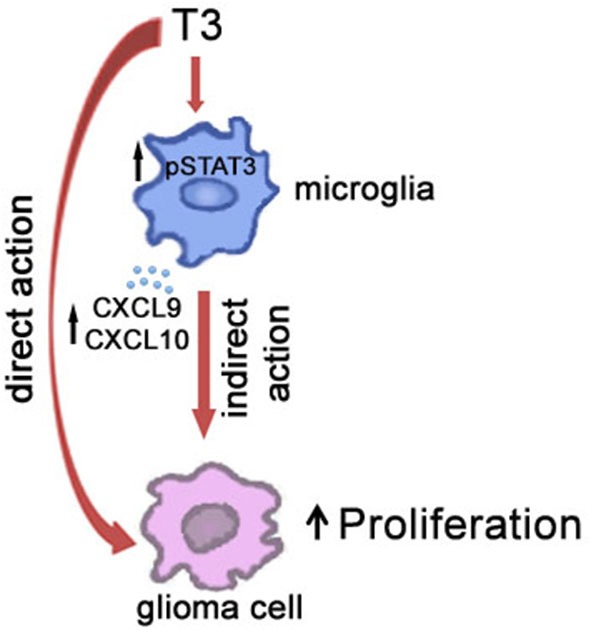
**Schematic illustration of the role of T3 in the cross-talk between microglia and glioma cells in the tumor microenvironment**.

The emerging recognition of the roles of microglia in health and disease has stimulated substantial efforts to define more clearly the regulatory mechanisms that control their functions. With respect to CNS pathological remodeling induced by dysregulation of plasmatic levels of THs, the characterization of the physiologic factors that regulate the establishment of the microglial/glioma network is challenging. It has been previously hypothesized that changes in the host stroma associated with hypothyroidism rather than a direct receptor-mediated action on the tumor cells may be responsible for THs-induced modulation of tumor growth (Martínez-Iglesias et al., 2009a,b). In this context, since THs are known to cross the blood-brain barrier and microglial cells are CNS targets of THs, it is reasonable to assume that T3, beside a direct modulation of brain cancer cell itself, influences the relationship of tumor cells with stroma cells (Figure 2). In particular, our suggestion that T3 indirectly promotes glioma growth through a modulation of microglia, deserves further consideration and may help to understand better the role of T3 dysregulations in brain tumorigenesis. Given the important clinical impact of glioma tumors, clariyfying T3-induced microenvironment regulations may open the field to significant advances in the identification of possible new strategies to cancer therapy thus translating the role of thyroid gland status into clinical cancer cell biology.

## Conflict of Interest Statement

The authors declare that the research was conducted in the absence of any commercial or financial relationships that could be construed as a potential conflict of interest.
